# Development of a Polysaccharide-Based Hydrogel Drug Delivery System (DDS): An Update

**DOI:** 10.3390/gels7040153

**Published:** 2021-09-27

**Authors:** Janarthanan Pushpamalar, Puviarasi Meganathan, Hui Li Tan, Nuraina Anisa Dahlan, Li-Ting Ooi, Bibi Noorheen Haleema Mooneerah Neerooa, Raahilah Zahir Essa, Kamyar Shameli, Sin-Yeang Teow

**Affiliations:** 1 School of Science, Monash University Malaysia, Jalan Lagoon Selatan, Bandar Sunway, Subang Jaya 47500, Malaysia; puviarasi@kingsley.edu.my (P.M.); c2dhlt@nus.edu.sg (H.L.T.); nuraina.dahlan@monash.edu (N.A.D.); 2 Monash-Industry Palm Oil Education and Research Platform (MIPO) & Tropical Medicine and Biology Platform (TMB), Monash University Malaysia, Jalan Lagoon Selatan, Bandar Sunway, Subang Jaya 47500, Malaysia; 3 School of Health Sciences, International Medical University, 126, Jalan Jalil Perkasa 19, Bukit Jalil, Kuala Lumpur 57100, Malaysia; ooi.liting@student.imu.edu.my; 4 Department of Medical Sciences, School of Medical and Life Sciences, Sunway University, Jalan Universiti, Bandar Sunway, Subang Jaya 47500, Malaysia; 16101248@imail.sunway.edu.my (B.N.H.M.N.); 18057877@imail.sunway.edu.my (R.Z.E.); 5 Department of Chemical and Environmental Engineering, Malaysia-Japan International Institute of Technology, Universiti Teknologi Malaysia, Kuala Lumpur 54100, Malaysia; kamyar@utm.my

**Keywords:** hydrogel, polysaccharides, drug, delivery systems, nanoparticles

## Abstract

Delivering a drug to the target site with minimal-to-no off-target cytotoxicity is the major determinant for the success of disease therapy. While the therapeutic efficacy and cytotoxicity of the drug play the main roles, the use of a suitable drug delivery system (DDS) is important to protect the drug along the administration route and release it at the desired target site. Polysaccharides have been extensively studied as a biomaterial for DDS development due to their high biocompatibility. More usefully, polysaccharides can be crosslinked with various molecules such as micro/nanoparticles and hydrogels to form a modified DDS. According to IUPAC, hydrogel is defined as the structure and processing of sols, gels, networks and inorganic–organic hybrids. This 3D network which often consists of a hydrophilic polymer can drastically improve the physical and chemical properties of DDS to increase the biodegradability and bioavailability of the carrier drugs. The advancement of nanotechnology also allows the construction of hydrogel DDS with enhanced functionalities such as stimuli-responsiveness, target specificity, sustained drug release, and therapeutic efficacy. This review provides a current update on the use of hydrogel DDS derived from polysaccharide-based materials in delivering various therapeutic molecules and drugs. We also highlighted the factors that affect the efficacy of these DDS and the current challenges of developing them for clinical use.

## 1. Introduction

Drug delivery is defined as the process of administering a pharmaceutical compound to achieve the targeted transport and release to specific therapeutic sites in humans and animals via various routes of administration, including oral, transmucosal, topical, inhalation, and intravenous routes. Although drug delivery has a long history dating back to when humans first ingested herbal remedies, most of the notable technological advances only began to take place in the 1970s. Since then, the advancement has not only focused on the targeted delivery of drugs, but also on maintaining a controlled release rate, achieving greater cellular uptake, and reducing the non-specific effects, all of which often require the drug to have a high shelf-life and stability [1,2,3,4]. To this end, the modern drug delivery systems (DDSs) play a crucial role in ensuring the above criteria are met. Among the DDSs, a polysaccharide-derived system has drawn considerable attention due to its numerous attractive characteristics [5].

Polysaccharides are carbohydrates consisting of repeating monosaccharide units linked by glycoside bonds. Polysaccharides can be isolated from cheap natural resources, such as algae (e.g., alginate), plants (e.g., starch, cellulose, and pectin), animals (e.g., chitosan, hyaluronic acid, and chondroitin), and microorganisms (e.g., pullulan, xanthan gum, and dextran) [6,7,8]. These polymers possess a wide range of physical, chemical, and biological properties which contribute to their high biocompatibility and bioactivity, and low enzymatic degradability. In addition, the presence of freely available functional groups such as carboxyl, amino, hydroxyl, and other hydrophilic groups allow drug conjugation and a series of chemical modifications which suit the desired applications [9,10]. However, limitations of DDS derived from natural polysaccharides compared to synthetic polymers such as polylactic acid (PLA), polylactic-co-glycolic acid (PLGA), and polyethylene glycol (PEG), have been recognized over the years [11]. These drawbacks include inconsistent properties due to different sources, uncontrolled uptake of water, low mechanical strength, and uncertain degradation patterns [9]. This has given rise to the development of DDSs using polysaccharide derivatives such as carboxymethylcellulose (CMC), as well as the subsequent formation of nanoparticle (NP)-based and hydrogel-based DDSs to overcome the abovementioned issues [12,13].

Over the years, micro- and nano-particulate DDS have gained interest owing to their unique characteristics such as a higher cell-penetrating capacity, target specificity, suitability for various types of administration routes, rich surface chemistry, and high flexibility in chemical modifications [14]. Examples of these DDS include microspheres and microcapsules. Microspheres are monolithic microparticles with a spherical shape, while microcapsules are microparticles with a core surrounded by other substances [15]. As the reduced size of DDS could contribute to the improved delivery of therapeutic molecules, the NP DDS have been introduced with added features of stimuli-responsiveness and sustained release [16]. On the other hand, hydrogel-based DDSs, which are mostly derived from polysaccharides, are also used as a popular vehicle due to their high biocompatibility, high biodegradation, and low immunogenicity [17]. Due to the advantages of NPs and hydrogel DDSs, both have been combined to generate various types and sizes of NP-hydrogel DDSs such as macrogels, microgels, and nanogels to maximize the therapeutic efficacy of DDSs [18].

In this review, we provide updates on the utilization of polysaccharides for hydrogel-based DDS construction. We discuss different types of therapeutic molecules delivered by these DDSs from recent studies and highlight the factors that could affect the efficacy of these DDSs, as well as the challenges in their development for clinical use.

## 2. Hydrogel-Based Drug Delivery Systems

The use of polysaccharides for the development of hydrogels became an area of interest due to their high biocompatibility and biodegradability. Other advantages include accessibility, cost-efficiency, and ease of surface modification through chemical means. Polysaccharides-based hydrogels are based on chitosan, starch, cellulose, pectin, alginate, psyllium husk, hyaluronic acid, guar gum, and their derivatives. Physical and chemical methods are used to crosslink the natural polymers to form beads, films, and disks. Chemicals crosslinkers (glutaraldehyde, epichlorohydrin, tripolyphosphate, and 1-ethyl-3-(3-dimethylaminopropyl)carbodiimide (EDC), and radiation, such as gamma and electron beams) are used to crosslink these natural polymers [19]. The drug carriers formulated from natural polymers are considered an ideal material to ensure the safety of patients due to their colonic enzymatic degradation into harmless by-products that are easily excreted from the body. 

Hydrogels are three-dimensional (3D) crosslinked polymer networks with a high swelling due to the presence of hydrophilic groups (e.g., -OH, -CONH^−^, and -SO_3_H). Hydrogels possess tunable physicochemical properties, low immunogenicity, ease of functionalization, and biocompatibility [20]. Polysaccharide-based hydrogels are highly desirable as they are intrinsically biodegradable and renewable, as well as abundant in nature [21]. Moreover, hydrogels can be engineered to encapsulate various therapeutic materials (e.g., small-molecule drugs, hydrophobic and hydrophilic drugs, growth factors, and inhibitors) and to protect labile drugs against degradation. The further development of hydrogels by incorporating nanomaterials (e.g., NPs, nanowires, nanotubes, and nanosheets) for the formation of hydrogel nanocomposites can be advantageous for DDSs, contributing to the increase of antibacterial activity, mechanical stability, and magnetic properties [18].

Hydrogel-based drug carriers differ in size (e.g., macrogels, microgels, and nanogels), architecture, and physical/chemical functions that dictate the release of drugs for targeted DDS. Macroscopic hydrogels (i.e., in situ-forming, microporous, and shear-thinning hydrogels) are suitable for transepithelial administration or injection, whereas hydrogel-forming microneedles have been widely used for transdermal drug delivery. Microgels are micrometer-sized hydrogel particulates that have shown promising potential for oral and pulmonary drug delivery. On the other hand, nanogels (10 to 100 nm) are suitable for systemic delivery. Hydrogels are attractive due to their ‘soft’ properties and high water content which are achieved either via physical or chemical crosslinking methods and allow them to be used for non-invasive delivery through injection. Physical crosslinking occurs via non-covalent interactions such as hydrogen bonding, hydrophobic–hydrophilic interactions, and ionic interactions. Typically, the physically crosslinked hydrogels manifest reversible sol-gel characteristics; rendering them sensitive to various factors such as polymer composition, temperature, ionic strength, and crosslinking initiators [22,23]. Although the preparative conditions for physical gelation are usually mild and avoid toxic reagents, hydrogels typically have poor mechanical properties. Therefore, physically crosslinked hydrogels would likely have a low stability in the harsh environment of body blood circulation [24]. Chemical crosslinking prevents the dissolution of polymer chains in aqueous media resulting in better gel stability compared to physically crosslinked hydrogels. The formation of covalent linkages between the polymeric chains increases the stability and integrity of the crosslinked networks. Typically, chemically crosslinked hydrogels are synthesized through the crosslinking of labile functional groups such as ester, carbonate, amide, anhydride, and phosphazene [23,24]. One of the common reactions is the Schiff-base reaction which forms imine bonds that are stable at physiological pH but labile at acidic pH [24]. Interestingly, both crosslinking strategies are tunable to fabricate smart hydrogels with functionalities (e.g., targeting ligands) that respond to physiological or extrinsic stimuli to trigger drug release. This section discusses the characteristics and functions of hydrogels at different sizes (i.e., macrogels, and microgels/nanogels), and the development of novel hydrogel-forming microneedles and stimuli-responsive hydrogels.

### 2.1. Macrogels

#### 2.1.1. In Situ-Forming Hydrogels

In situ-forming hydrogels offer easy administration via minimally invasive injection and the accurate dose delivery of therapeutic drugs in a controllable manner. The synthesis of in situ-forming hydrogels is categorized as non-covalent, due to temperature, pH, ionic, and self-assembly; and covalent, due to crosslinking light or chemical/enzyme precursors [25]. Non-covalent crosslinking induces the formation of secondary bonds (e.g., hydrogen bonding, and electrostatic and hydrophobic interactions), triggering the polysaccharides to undergo a sol-gel transition into semi-solid hydrogels via in situ gelling systems that respond to physiological stimuli (e.g., change of physiological pH, body temperature, and biological fluid’s ionic strength). This approach is ideal for the delivery of small-molecules and sensitive drugs, as it avoids using harmful chemical agents [26]. Yin and colleagues developed thermo-responsive in situ-forming hydrogels via physical crosslinking with a rapid gelation time of 15 s initiated closer to body temperature. The drug-loaded hydrogels show the sustained release of NPs co-loaded with indomethacin (IND) and methotrexate (MTX) over 72 h for the treatment of rheumatoid arthritis [27]. In this instance, the gelation rate of physical crosslinking is influenced by polymer and drug compositions, as well as collective physiological responses. The delayed gelation in physiological environments may cause a burst-out effect due to prolonged exposure in tissue exudates, causing rapid clearance and reducing drug bioavailability. The fabrication of in situ hydrogels using the chemical crosslinking method enhances the mechanical properties of hydrogels. This method utilizes chemical crosslinkers (e.g., genipin, formaldehyde, glutaraldehyde, and epoxy compounds) to react with crosslinkable functional groups of the polysaccharides [28,29]. For instance, the primary amino groups (-NH_2_) of chitosan and gelatin were co-crosslinked by β-glycerophosphate and genipin forming in situ hydrogels for intraocular drug delivery [29]. Recently, ‘click’ reactions such as Diels-Alder and thiol-ene reactions showed promising potential to produce in situ-forming hydrogels under mild preparative conditions [28,30].

#### 2.1.2. Macroporous Hydrogels

Macroporous hydrogels have been widely used to deliver therapeutic drugs, genes, or cells. In regenerative medicine (e.g., tissue engineering and wound healing), macroporous hydrogels can be ideal bio-hydrogels that protect cells and other bioactive materials against stresses during implantation and later promote cellular integration into the host tissues. The large and interconnected macropores enhance gaseous and nutrient exchanges to support cell infiltration and growth, thereby accelerating healthy tissue regeneration. In addition, macroporous hydrogels can load a very high amount of bioactive materials (e.g., drugs, growth factors, cytokines, and nanoparticles, etc.) attributed to their high porosity. Inevitably, these macropores are essential to fabricate effective drug carriers for targeted and localized delivery [31]. Cryogelation, particulate leaching, lyophilization, gas foaming, additive manufacturing (3D printing), freeze-thawing, and microgel assembly methods have been used to fabricate macroporous hydrogels. These techniques generally avoid harmful chemical agents or high temperatures, making macroporous hydrogels ideal to mimic the extracellular matrix (ECM) of native tissues [32,33]. Most recently, lyophilized macroporous D-galactose-based hydrogels exhibited a pore size ranging from 4 to 20 μm and the sustained release of gentamicin (hydrophilic drug) up to 92% within 72 h [34]. Although injectable drug-loaded hydrogels can be administered using non-invasive methods, they possess poor mechanical stability and lack porosity to efficiently entrap therapeutic materials, hence compromising their continued clinical use. Meanwhile, macroporous hydrogels exhibit excellent mechanical properties, porosity and interconnectivity permitting better control over the encapsulation and release of therapeutic materials [35,36]. Ideally, injectable macroporous hydrogels would possess macropores with sizes ranging from 50 to 300 μm to facilitate various cell adhesion and migration, including mammalian cells [37]. Combining the advantages of both injectable and macroposity properties would increase the drug carrier’s potential as a DDS. For example, fabrication of gelatin-based injectable macroporous hydrogels via the microgel assembly method resulted in higher cellular infiltration and the controlled release of platelet-derived growth factors, which enhanced cell proliferation [36].

#### 2.1.3. Shear-Thinning Hydrogels

**Figure 1:**
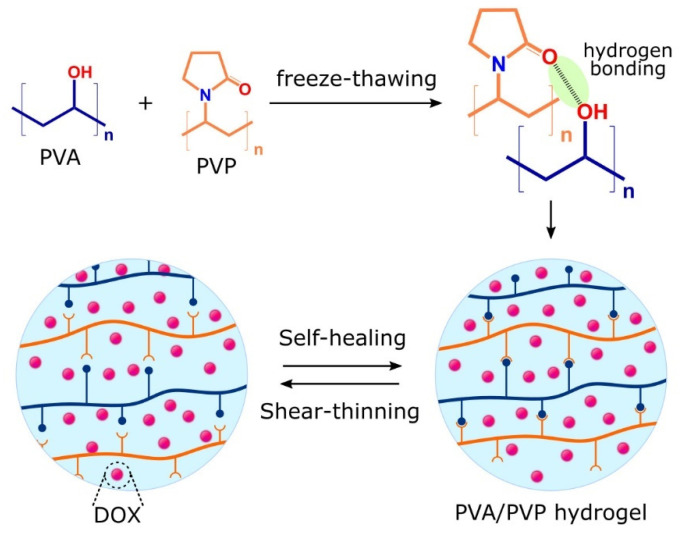
Illustration of shear-thinning and self-healing properties of doxorubicin (DOX)-loaded poly(vinyl alcohol)(PVA)/poly(vinyl pyrrolidone (PVP) hydrogels migrated along biomimetic microcapillaries model.

Hydrogels with shear-thinning properties undergo reversible gel-sol transition upon the application of shear stress. Interestingly, shear-thinning properties allow for the homogeneous encapsulation of drug payloads and smooth injection without the clogging of the injection needle by hydrogels returning to their original viscosity and shape once the mechanical load (shear stress) is removed [38]. Shear-thinning hydrogels are increasingly used in DDSs owing to their ability to conform to the shape of the injection cavity, thus maximizing contact with the targeted tissues/organs for localized drug delivery. Recently, Prasad et al. studied the shear-thinning properties of their hydrogels which migrated along constricted microchannels mimicking human microcapillaries. In their study, the hydrogels deformed and retained their original shape upon the cessation of a high shear, expressing both shear-thinning and self-healing properties, as illustrated in Figure 1. Furthermore, the hydrogels exhibited a longer retention of doxorubicin during migration with sustained drug release [39]. In this case, both shear-thinning and self-healing properties offer many advantages, including enhanced mechanical properties and reduced embolization risk in the systemic circulation compared to their counterpart, in situ-forming hydrogels [38]. Additionally, Gharaie et al. reported smart shear-thinning hydrogels with pH-responsive characteristics. They found that the hydrogels showed a significant swelling rate at pH 9.18, followed by high drug release presenting a sustained release characteristic ideal for treating wounds [40].

### 2.2. Microgels and Nanogels

#### 2.2.1. Synthesis and Characteristics of Microgels and Nanogels

The particulates of hydrogels on the size scale ranging from 10 nm to 5 μm, known as microgels or nanogels, are of growing interest for the delivery of specific therapeutic drugs to specific sites in the body [41]. In general, microgels less than 5 μm in diameter are suitable for oral and pulmonary delivery, while nanogels with a 10–100 nm diameter can be used for systemic drug administration. The advantages of microgels/nanogels for polymer-based DDSs include the stability of the interior network structure, a rapid response time, a large surface area for multivalent bioconjugation, and a high ability to retain water and bioactive drugs within their 3D network structure [42]. According to Oh et al., synthetic nanogels are primarily synthesized by the heterogeneous polymerization of hydrocarbon-based hydrophilic or water-soluble monomers in the presence of multifunctional crosslinkers [43].

**Figure 2:**
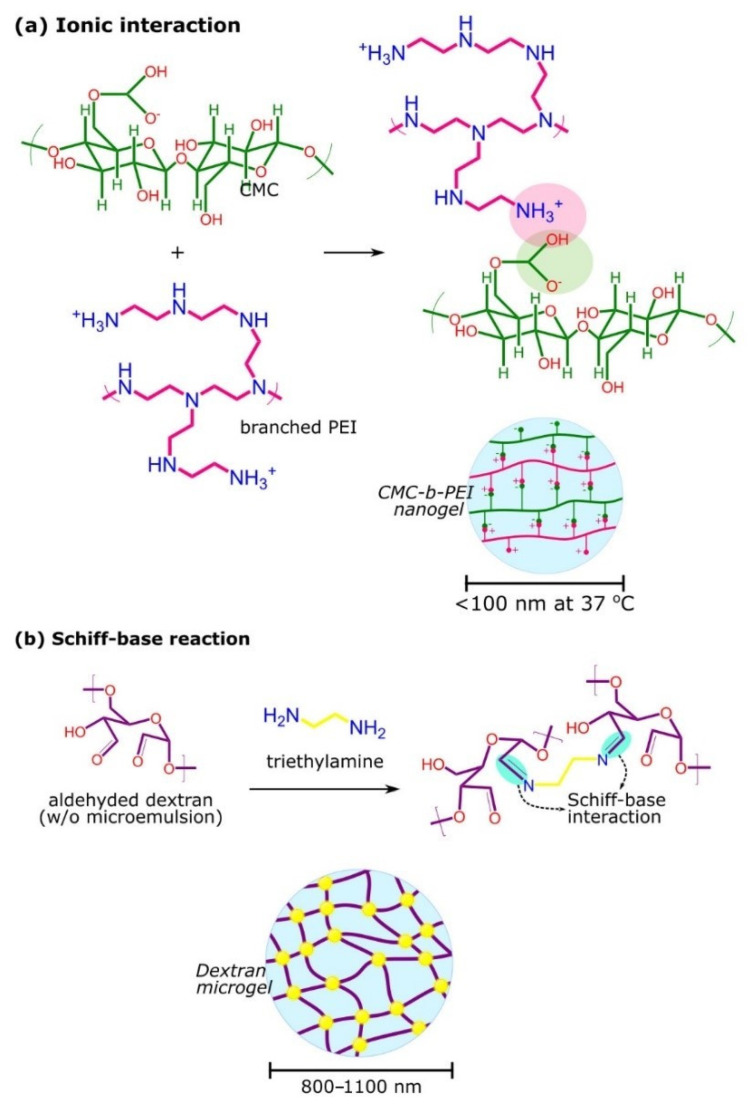
Examples of microgels/nanogels crosslinking mechanisms. (**a**) carboxymethylcellulose(CMC)-branched poly(ethyleneimine)(bPEI) nanogels obtained via ionic interaction and (**b**) formation of dextran microgels via Schiff-base reaction as pH-responsive drug vehicles.

Microgels/nanogels are formed by physically or chemically crosslinked polymer chains in micro- or nano-scale size (Figure 2). For physical crosslinking, this usually occurs through non-covalent attractive forces, including hydrophilic–hydrophilic, hydrophobic–hydrophobic, and ionic interactions, and/or hydrogen bonding. The properties of the polymer highly depend on several factors, such as polymer composition, temperature, ionic strength, and the crosslinking agent used [44]. For example, Nakai et al. synthesized a physically crosslinked nanogel based on the self-assembly of cholesteryl-group-bearing hyaluronic acid. In this study, injectable hydrogel was formed by the salt-induced association of the hydrogel [45]. In another study, poly(N-tert-butyl acrylamide) side chains were grafted on the methylcellulose backbone to form self-assembling nanogels through hydrophobic interactions. The nanogels released the entrapped dexamethasone slowly over several weeks, which showed its potential for ocular drug delivery [46]. Recently, Zhou et al. described the preparation of the self-assembled sodium alginate-chitosan nanogel, based on the interaction of the positive charge of carboxymethyl chitosan and the negative charge of sodium alginate [47]. Although the preparative conditions for physical gelation are usually mild and avoid the use of toxic reagents, the gels typically have poor mechanical properties that eventually cause low stability in the harsh environment in body blood circulation [24]. 

In contrast, chemical crosslinking prevents the dissolution of polymer chains in aqueous media. This is attributed to the presence of covalent linkages on the polymer chains that increase the stability and integrity of the structures. For instance, Wang et al. synthesized a microgel by crosslinking chitosan and gelatin with succinimide-end PEG, which exhibited a sustained release of folic acid within 72 h, at physiological pH and temperature [48]. Furthermore, no significant change in the dimension of the microgels was observed for 7 days, indicating the stability of the microgels [48]. To make the chemically crosslinked hydrogels biodegradable, crosslinks with labile bonds such as ester, carbonate, amide, anhydride, and phosphazene are usually introduced [49]. In a study by Su et al., microgels were formed via Schiff-base linkages between aldehyde dextran and ethylenediamine in a water-in-oil microemulsion, as shown in Figure 2b [50]. As the Schiff bases linkages are hydrolyzable and less stable at an acidic pH, the microgels exhibited a unique hydrolysis profile in tumors [50]. In another study, carboxymethyl starch microgels with various crosslinking densities were synthesized using sodium trimetaphosphate. The microgels were reported as viscoelastic solids when the storage modulus was higher than the loss modulus over the frequency range of dispersions with the polymer at concentrations of 3% (*w/w*) at 25 °C [51].

#### 2.2.2. Use of Microgels or Nanogels as Drug Delivery Systems

The application of nanogels in DDSs is highly advantageous, owing to the stable interior 3D networks for higher drug payloads, rapid response times, and larger surface area for multivalent bioconjugation. Microgels are favorable for oral and pulmonary drug delivery as these particulates can avoid fast renal clearance, sequestering by the spleen, and the phagocytosis by macrophages [52]. In a previous study, the aerodynamic size of the nanoparticle-inside-microgel (nano-in-microgel) system allowed deep pulmonary drug delivery while preventing the rapid clearance by alveolar macrophages. The microgel underwent enzymatic degradation releasing therapeutic nanoparticles to treat acute and chronic pulmonary diseases such as asthma, cystic fibrosis and chronic obstructive pulmonary disorder [52]. Owing to the size of nanogels, drug-loaded nanogels could easily reach the smallest capillary vessels and penetrate tissues either through paracellular or transcellular routes. Moreover, nanogels could enhance the pharmacological effect of small-molecule drug and a stability against chemical or enzymatic degradations [52]. The fabrication of nanogels using advanced technologies such as microfluidic-assisted methods allows for the controlled and large-scale production of nanogels [53]. Nanogels with a narrow size distribution are fabricated by controlling microfluidic conditions such as chip design, rheological properties, and tuning of the flow rate. The robust fabrication strategy enables high control over the nanogel’s properties (e.g., size, surface changes, and polydispersity), payload delivery and release mechanism [53]. In one study, alginate nanogels were fabricated using the microfluidic method. It was found that the uniform and monodisperse nanogels can be tuned by adjusting the flow rate ratio, thus controlling the release profile of payloads and avoiding premature (burst) release [53].

Over the years, polysaccharides-based microgels/nanogels have attracted considerable attention in drug delivery applications as they are intrinsically biodegradable and renewable, as well as abundant in nature. It is known that hydrogels between 0.5–10 μm can be phagocytized by macrophages, while hydrogels smaller than 10 nm can be efficiently removed by the kidney [54,55]. As microgels/nanogels derived from polysaccharides are hydrophilic in nature, they have been widely studied as the drug carriers for hydrophilic drugs. Recently, layer-by-layer (LBL) polyelectrolyte-coated alginate microgels were synthesized for the DDS. The LBL deposits of poly(allylamine)/poly(sodium 4-styrenesulfonate) (PAH/PSS) on alginate microgels acted as a diffusion barrier for sustaining the release of minimal hydrophilic drugs. The barrier mechanism worked by increasing the diffusion pathway and molecular interaction potential between drug molecules and the bilayers. The study reported that the novel microgels successfully sustained the release of sodium benzoate and zosteric acid for up to 72 and 120 h, respectively [56]. Yurkshtovich et al. demonstrated the ability of starch phosphate microgels to encapsulate interferon-alpha 2b and aimed to deliver the therapeutic agent for cancer or viral infection treatments [57]. Furthermore, Zhang et al. developed the self-stabilized hyaluronic acid nanogels for the codelivery of doxorubicin and cisplatin to treat osteosarcoma. Cisplatin acted as the crosslinker and ancillary anti-carcinogen. As a result, the premature release of doxorubicin was avoided, and a synergistic anti-tumor effect was achieved [58].

Interestingly, microgels/nanogels can be promising candidates for delivering poorly soluble drugs, given that the polymer structures are appropriately engineered [59]. For example, Sawada et al. developed a new self-assembled natural polysaccharide nanogels using xyloglucan (XG) [60]. The reaction between XG and cholesterol N-(6-isocyanatohexyl) carbamate resulted in cholesterol-bearing XG (CHXG) molecules. These molecules self-assembled in water due to the association of the introduced cholesteryl group to form nanogels with a diameter of 100 nm. They also reported that CHXG nanogels could be complexed with paclitaxel yet remained in the nanosized range, demonstrating the CHXG nanogels’ potential as drug carriers. In another study, MTX was conjugated with chondroitin sulfate (CS), forming a self-assembly nanogel for cancer drug delivery. MTX-CS nanogels are reported to improve drug solubility, enhancing the anti-tumor effect while lowering the side effects of MTX [61]. Other than modifying the polymer structures, hybrid nanosystems can also be used. Recently, Fiorica et al. synthesized the hyaluronic acid/polyaspartamide-based double-network graphene oxide-containing nanogels for colorectal cancer treatment. The presence of graphene oxide allowed the loading of a large amount of irinotecan (33.0% *w/w*) and contributed to the hyperthermic properties of the nanogels when irradiated with a near-infrared laser beam [62]. A chitosan containing magnetite nanogels was loaded with an anti-cancer drug, 5-fluorouracil, by encapsulation and adsorption processes. The drug release from the nanogel was 46% and 100% for adsorption and encapsulation processes in 6 h, respectively [63].

### 2.3. Hydrogel-Forming Microneedles

**Figure 3:**
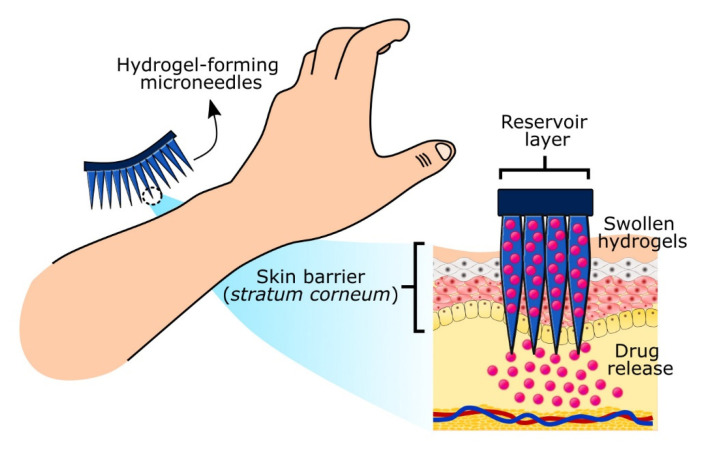
Transdermal drug delivery of highly responsive hydrogel-forming microneedles.

Microneedle technology is an emerging technology enabling transdermal drug delivery by puncturing micro-sized needles through the stratum corneum (skin barrier). These microneedle arrays create microscopic pores delivering drugs to skin microcirculation and achieving systemic delivery through transdermal administration. Hydrogel-forming microneedles (hydrogel-MNs) swell to form transient microscale delivery channels to release drugs without leaving polymer residues behind while minimizing skin damage and bleeding. The drug delivery mechanism involves the swelling of the hydrogel-MNs, releasing payloads loaded either in the reservoir layer or within the crosslinked polymers that diffuse into the dermal microcirculation (Figure 3). Notably, hydrogel-MNs show the promising administration of high-dose drugs (e.g., metformin hydrochloride, doxorubicin, trametinib, rifampicin, isoniazid pyrazinamide, and ethambutol) and controlled drug release [64,65,66]. Hydrogel-MNs address several challenges associated with other drug administration routes (e.g., oral, pulmonary, and parenteral), such as minimizing gastrointestinal side effects, rapid clearance and increasing patient compliance due to pain-free administration. Typically, the height of microneedles ranges between 50 to 900 μm, allowing them to penetrate the dermis layer while being short enough to reach the dermal nerves and blood vessels located deeper into the dermis layer as illustrated in Figure 3 [64]. Alternatively, hydrogel-MNs can also be used as biosensors collecting biomarkers from the interstitial skin fluid (ISF). They represent alternative sampling strategies compared to invasive sampling approaches (e.g., blood draws, skin, and other tissues biopsies), which can cause patient discomfort and anxiety. In this instance, microneedle technology is highly demanded as an advanced point-of-care system for frequent monitoring (e.g., blood glucose level) and diagnostic pathology, including cancer [67,68]. For example, glucose-responsive hydrogel-MNs served as a blood glucose sensor and self-regulated drug/insulin delivery governed by the high sensitivity towards elevated glucose concentration. Such multifunctional hydrogel-MNs can serve as intelligent artificial devices for advanced diabetes management [66,68].

### 2.4. Stimuli-Responsive Hydrogels

**Figure 4:**
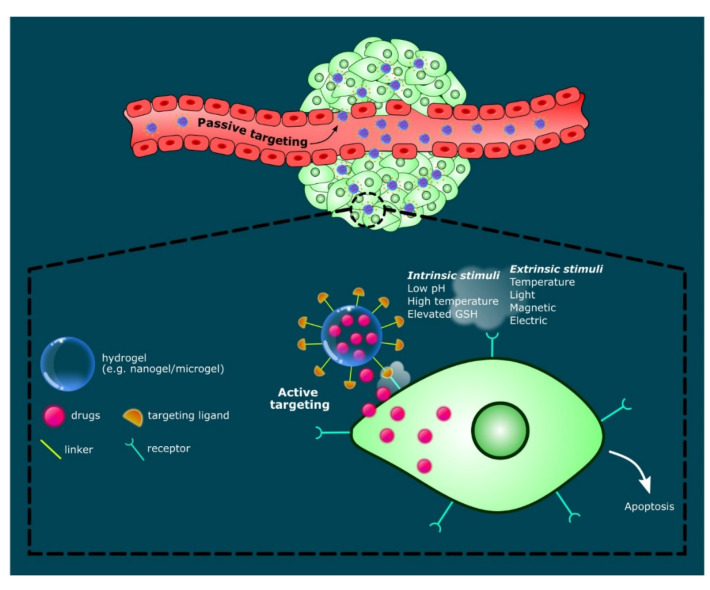
Schematic illustration of stimuli-responsive hydrogels, targeting mechanisms (i.e., passive and active targeting) and intracellular anti-cancer drug release leading to cell apoptosis.

The functional groups of polysaccharides can be functionalized to form hydrogels that respond to intrinsic or extrinsic stimuli such as pH, ionic strength, temperature, magnetic, and electric field, triggering the release of bioactive materials (e.g., hydrophilic, hydrophobic and lipophilic drugs). In particular, hydrogels express high sensitivity towards changes in temperature and pH. Therefore, these properties have been widely utilized to design innovative drug carriers for cancer therapy. These drug-hydrogel systems are sensitive to the high temperature and the acidic pH of tumor microenvironments, causing the cleavage of polymer networks/linkages resulting in swelling or degradation, hence releasing drugs in a targeted manner [69,70]. For example, Zhou’s group designed hybrid microgels releasing multiple drugs upon activation by the special conditions of tumor microenvironments (i.e., acidic pH, high temperature and elevated glutathione (GSH) level), which can be an effective strategy for combination therapy [70]. Like other biomaterial particles, microgels and nanogels can provide both passive and active targeting strategies, particularly in cancer therapy. In passive targeting, drug-loaded microgels/nanogels with a specific size, shape, and enhanced permeation and retention (EPR) affect circulate through the bloodstream before reaching and diffusing through the leaky vasculature of the targeted tumor. Additionally, functionalized hydrogel particulates with targeting ligands enable the active targeting of overexpressed tumor receptors to induce drug release, as illustrated in Figure 4. Such complex drug-hydrogel systems demonstrate a promising platform for the non-invasive delivery of accurately dosed drugs. Karzar Jeddi and Mahkam demonstrated the pH-responsive behavior of magnetic spherical carboxymethylcellulose-alginate-chitosan nanogels [71]. At simulated conditions of gastric (pH 1.2), intestinal, (pH 7.4) and cancer cell environment (pH 5.8), the nanogels released approximately 90% of dexamethasone, especially in simulated cancer cell microenvironments. The drug release reduced significantly at pH 7.4 (26.2%) and pH 1.2 (29.5%), respectively [71]. The stimuli-responsive drug-hydrogel systems proved promising for various biomedical applications such as in cancer therapy, wound healing, and tissue engineering. 

## 3. Drugs and Inhibitors Delivered by Hydrogel-Based Delivery Systems 

### 3.1. Delivery of Genetic Materials

Gene therapy that involves the delivery of genetic material such as antisense oligodeoxynucleotides (ODNs), plasmid DNA (pDNA), siRNAs (small interfering RNAs), and microRNAs (miRNAs) has been recognized as a potential method for the treatment of cancer, neurodegenerative disorders, and viral infections [72,73]. There are various types of gene delivery systems designed for the restoration of a specific gene function or to knock down the expression of a specific gene. The applications of non-viral vectors made of cationic carriers such as lipids, liposomes, and peptides have been studied due to their more substantial capacity to carry genetic material. Microgels/nanogels have also been studied as non-viral carriers due to their low toxicity and immunogenicity [73]. For example, cationic biodegradable dextran microgels have been synthesized by the emulsion polymerization and loaded with siRNA. The siRNA-loaded microgels escaped the exposure of ultraviolet light and/or free radicals, preventing the integrity of siRNA from being affected. Besides, high siRNA encapsulation efficiency can be obtained based on the number of cationic groups incorporated in the microgel network. In addition, the release-time of the encapsulated siRNA can be controlled by varying the crosslink density of the microgels [74]. In another study, a glycol chitosan-based nanogel was synthesized through the chemical grafting of hydrophobic chains onto a hydrophobic backbone to form an amphiphilic polymer that was able to self-assemble in an aqueous environment. As folate receptors are highly expressed in some tumors, folate was conjugated to glycol chitosan by PEG. The results showed that the nanogel-bearing folate enhanced the internalization of the nanogel following the folate receptor-mediated endocytosis. Besides, the nanogels were shown to complex with siRNA efficiency, showing a potential vehicle for siRNA delivery [75]. In a study by Yang et al., CMC was complexed with branch-type cationic poly(ethyleneimine) (bPEI), to form nanogel for the encapsulation of plasmid DNA [76]. Based on the results, the cytotoxicity of bPEI was lowered when it was in complex with CMC. The CMC nanogel complexed with bPEI which improved the cellular uptake of DNA, as well as gene transfection in stem cells [76]. A silane-crosslinked PEI network and a pH-responsive poly(2-(hexamethyleneimino)ethyl methacrylate) (PC7A) network was used to make double-network nanogels. It was also found to facilitate the release of DNA and endosomal escape at low pH [77]. An oral delivery of nucleic acids in a TAC6 nanogel to the colon was explored, using caveolae-mediated cellular uptake. The protein inhibitor of activated STAT1 (PIAS1) is a therapeutic target for inflamed bowel disease as it has the ability to control NF-KB inflammatory signaling. It was shown that using TAC6-derived nanogel effectively deliver PIAS1 and decrease the inflammation of the gut in a murine colitis model [78]. Other nanogels used for the delivery of miRNA delivery include nanobody conjugated DNA strands loaded onto the surface, forming a nanobody-functionalized nanogel which is used for tumor-targeted miRNA delivery for anti-tumor treatment. The study showed that using a nanobody-functionalized nanogel resulted in increased accumulation of miRNA at the tumor site, mediating the anti-tumor effect [79].

### 3.2. Delivery of Peptides or Proteins Using Stimuli-Responsive Hydrogels

Proteins and peptides show promising therapeutic potential; however, they often present limitations such as low stability, low bioavailability, and high immunogenicity [80,81,82]. Some of these issues can be solved by using hydrogels as the DDS. For example, Theune et al. reported that the hydrophilic, dendritic polyglycerol in the nanogel was able to stabilize the particle size as a crosslinker and, with the co-polymerization of poly(N-isopropylacrylamide) (NIPAM) and poly(N-isopropylmethacrylamide), it was able to adjust the volume phase transition temperatures to its desired range at the physiological condition of 34–37 °C [80]. This allowed the release of the encapsulated protein when there was a temperature change within the range [80]. A nisin-loaded antimicrobial dual responsive nanogel, made of poly(L-lactide)-graft-chondroitin sulfate (PLLA-g-CS) was shown to release and exert potent antibacterial activity depending on the pH and temperature of release media. This nanogel could be used for the pH- and temperature-sensitive delivery of proteins or peptides [81]. An embedded polymeric nanogel was found to preserve the activity of cytochrome c, an activator of cell apoptosis. The nanogels (CytoC/aNGs) increased the cellular uptake of cytochrome c and improved the intracellular delivery to the targeted sites to induce apoptosis [82]. In another study, pH and glucose dual-responsive biodegradable nanogels were constructed and showed the release of the encapsulated proteins [83]. These nanogels were highly dependent on glucose or pH concentrations, where low amounts of proteins were released in physiological pH or under healthy blood glucose level, while the rapid release was seen at lower pH or under diabetic blood glucose level [83]. Massi et al. generated a temperature-sensitive nanogel that responded to matrix metalloproteinase-7 (MMP-7) and with the addition of N-cyclopropylacrylamide (NCPAM) into NIPAM-based copolymer, it allowed the encapsulation of protein cargo and nanogel-crosslinking at slightly elevated temperatures [84].

### 3.3. Delivery of Drugs

Drugs used in chemotherapy are known to have a poor stability, low bioavailability, high toxicity, and non-specificity. Many of these issues can be solved using hydrogels as the DDS [14]. A study by Li et al. demonstrated a better cellular uptake and improved cytotoxicity in acid-degradable nanogels which released the DOX drug into the multicellular tumor spheroids and inhibited growth compared to non-sensitive nanogels [85]. The acid-degradable nanogels were stable in the physiological environment and could be degraded in a mildly acidic environment [85]. A study reported that hyaluronic acid (HA):sucrose nanogels acted as potent drug carriers for hydrophobic anti-cancer drugs and prolonged the drug release due to the chemical functionality of drug conjugation, adsorption, porosity, and degradability [86]. In another study, the hypoxia-responsive zwitterionic poly(phosphorylcholine)-based nanogel (^H^PMPC) was able to degrade rapidly in a hypoxic environment and led to the release of the loaded drug which then inhibited the tumor both in vitro and in vivo [87]. More interestingly, the nanogels could pass through the blood–brain barrier (BBB) without any external assistance, as the nanogels were able to mimic the cell membrane of phosphorylcholine polymers leading to the accumulation of nanogels at the desired site [88]. The promising nanogel carriers, harmine nanocrystals, were generated by Huang et al. for nose-to-brain delivery of poorly soluble drugs [89]. The study showed a 25-fold increase in bioavailability in the brain through the intranasal route compared to the oral route [89]. A pH-triggered charge-reversible chitosan-polypyrrole nanogel had a good cytocompatibility and excellent protein resistance, as well as a high DOX loading efficiency. This self-adaptive charge-switching DOX-loaded nanogel exhibited a prolonged blood circulation time, enhanced tumor acceleration, better penetration, and a high uptake in tumor cells, making it an ideal candidate to deliver the chemotherapeutic drugs [90]. Qu et al. [91] developed a prodrug nanogel made from methacrylic acid (MAA), CPT monomer (CTPM), and N-N’-methylenebisacrylamide which was named as P(CPT-MAA). This nanogel was a redox/pH dual-dependent nanogel, regulated by concentrations of glutathione and pH, which inhibited tumors both in vitro and in vivo under the desired conditions [91]. In another study, a supramolecular polymer nanogel was used to deliver an anti-inflammatory drug, Dexamethasone, dermally. This drug was released from the nanogel via a host–guest molecular recognition [92]. A study demonstrated that bovine serum albumin-gum arabic aldehyde (BSA-GAA) containing 5-fluorouracil responded to the pH changes and the high level of drugs were released at lower pH and inhibited the growth of MCF-7 breast cancer cells [93]. Other than drug delivery for cancer therapy, vancomycin-loaded DNA nanogels were made to deliver antibiotics. This nanogel showed a constant release of vancomycin over the course of 24 h, as well as synergistic anti-inflammatory and antibacterial activities [94]. Table 1 summarizes the inhibitors and drugs delivered by polysaccharide-based hydrogels DDSs.

## 4. Factors Affecting the Efficacy of Hydrogel Drug Delivery Systems

### 4.1. Size of Nanoparticles

The size of polysaccharide-based NPs is particularly attractive to determine the efficiency of drug encapsulation and the release profile. In addition, the size of NPs is predominantly essential to determine the ability of a drug nanocarrier to localize and bind to the targeted cell membrane, and for the cellular interaction, including cellular uptake, half-life, and biodistribution of the NPs to take place [95,96]. The molecular weight of the polymer used for the formation of NPs can profoundly influence the size of the synthesized NPs [97]. Karimi et al. demonstrated that the increased size of magnetic chitosan NPs crosslinked with ҡ-carrageenan was observed by increasing the molecular weight of chitosan [97]. The same study then reported the increased loading efficiency of sunitinib following the increase of chitosan molecular weight and the size of NPs. However, the release rate of sunitinib decreased due to the increased molecular weight of chitosan and larger size of NPs [97]. In another study, Unsoy et al. reported the decreased size of chitosan-coated magnetic NPs following the decreased amount of ammonium hydroxide (NH_4_OH) used during the synthesis [98]. The same study also demonstrated the increased release of loaded doxorubicin from the smaller size of chitosan-coated magnetic NPs. A similar trend in findings was also reported in a study by Bruinsmann et al. [99]. Another study by Tran et al. generated a smaller nanoplex of chitosan and curcumin through the milifluidic method and showed a higher supersaturation which improved the bioavailability of the DDS compared to the larger nanoplex [100].

### 4.2. Shape of Nanoparticles

Other than size, the shape geometry of NPs is also a factor that could affect the pharmacokinetics and pharmacodynamics of drugs, the transport properties in fluids, and the adhesion kinetics of the NPs to target cells [101]. While NPs were conventionally synthesized in spherical shapes, recent NP engineering has enabled the generation of NPs in different shapes including rods, prisms, cubes, stars, and disks [102]. Different methods that could produce NPs in controlled shapes are lithography-based, membrane stretching, and self-assembly methods [103]. In the study reported by Agarwal et al., asymmetrical NPs were found to enhance the penetration and distribution of NPs in the solid tissues and tumor. Interestingly, nanodiscs showed a higher accumulation in the tumor spheroids compared to nanorods of similar size [104]. This suggested that NPs in different geometries could interact with cell membranes differently and might exert different biological activities. With the advent of nanotechnology, modifications can be made on native cellulose fibers to manufacture nano-celluloses including cellulose nanowhiskers (CNWs), nanocrystals and nanofibers [105,106,107,108,109]. The rod-like CNWs are believed to be a promising DDS owing to their nano-dimensions, large surface area, hydrophilic properties, and high biocompatibility and biodegradability [110].

### 4.3. Hydrogel Swelling Ratio

Hydrogels have the capacity to swell to hundreds or thousands of times their own weight [111]. The swelling ratio is indicated by the amount of water absorbed and retained the hydrogel matrix [112]. The ability of hydrogels to absorb water depends on the composition of hydrophilic functional groups, the structure of the polymers, the interaction between polymers and solvent, and the crosslinking degree [113]. The equilibrium swelling ratio is reached when the balance occurs between the osmotic forces of the functional groups and the restrictive forces of the higher ordering of the polymer [114,115]. The swelling ratio is determined by the following equation [116]:

(1) $$\mathrm{Swelling}\;\mathrm{percentage}\;\left(\%\right)=\left(\frac{m_{s}-m_{d}}{m_{s}}\right)\times 100$$

where *m_s_* and *m_d_* are the mass of swollen and dried hydrogel samples, respectively.

**Figure 5:**
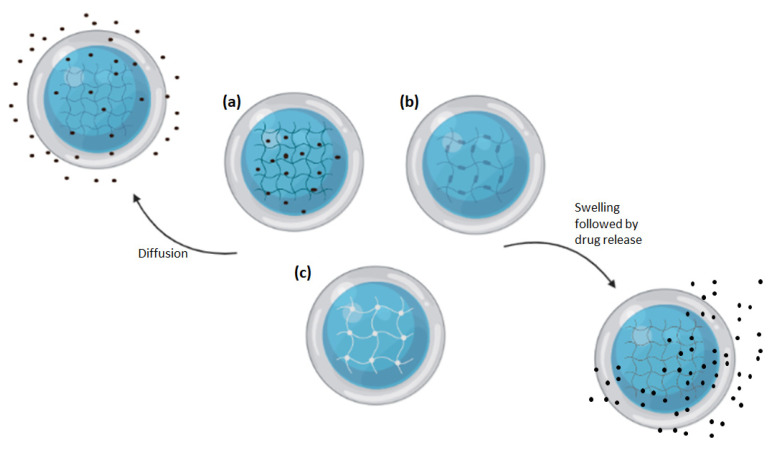
Illustration of drug loading and releasing mechanisms of hydrogels. (**a**) Hydrogel-containing drug dissolved within its matrix. (**b**) Hydrogel-containing drug covalently attached to the polymer mesh. (**c**) Hydrogel-containing drug entrapped within its mesh. Drugs can either be released through diffusion or the swelling of the hydrogel which causes enlarged pore and mesh size.

The swelling ratio of hydrogel is closely related to its release kinetics which drastically affect the diffusion of drugs [116]. As hydrogels are made of polymeric networks, drugs can become entrapped in the polymer matrix complex or attached to the polymer either via covalent bonding or physical attraction via a hydrogen bond, an ionic bond, or hydrophobic interaction (Figure 5). These properties allow hydrogel to carry a concentrated amount of drugs to their target site and release the drugs through diffusion, swelling, chemical control, or stimuli [116].

## 5. Challenges in Hydrogel Drug Delivery System Development

While polysaccharides possess a large number of advantageous properties making them suitable in the development of delivery systems for various types of drugs, they are also associated with disadvantageous properties. For example, chitosan alone has low solubility and is extremely pH sensitive, limiting the chemical modifications that can be carried out as well as the routes of administration that can be used. Both oral and mucosal administrations are not ideal with chitosan as the pH sensitivity in acidic conditions results in rapid degradation in the stomach, while the low solubility in water limits the desirable mucoadhesive properties, resulting in poor absorption through mucosal sites [117]. Other polysaccharides such as pectin and guar gum are also associated with limitations, such as poor mechanical stability [118,119]. However, many of these limitations can be addressed through surface modification or incorporation of other molecules. Therefore, the major limitations in the development of DDSs actually lie in the developmental stages, as many factors and requirements need to be taken into consideration. The requirements of an effective DDS that need to be accommodated for, include maintaining both structural stability, the biological properties of the drug, and the polysaccharide; maintaining a small size distribution; high encapsulation efficiency and loading capacity; low levels of aggregation; release profiles suitable for the intended use, and the display of no cytotoxic effects [120]. Furthermore, biodegradability, high biocompatibility, gel forming capacity, high polysaccharide stability, and good swelling potential are also considered essential for an effective hydrogel DDS [120]. For the development of an appropriate hydrogel DDS with ideal biological properties, not only do all the different requirements need to be identified, but they also need to be successfully implemented in the synthesis process and accurately characterized.

In addition to the general requirements, the methodology needs to be optimized to meet the ideal physicochemical properties of the DDS. Depending on the intended use, which includes the nature of the therapeutic agent being encapsulated, the target delivery site, the ideal hydrogel size, shape, degradation rate, and response to surrounding stimuli, as well as bioavailability need to be achieved. Furthermore, due to the highly variable molecular weights and surface chemistry of polysaccharides, the development of polysaccharide-based DDS is more difficult than the development of synthetic polymer-based DDS [121]. One of the physiological properties that determines both DDS cellular uptake and bioavailability is the size of the hydrogels [122]. While numerous methods have been identified to optimize the production of nanogels with a desired size range, as well as a uniform distribution of size, the propensity of polysaccharide-based nanogels to form crosslinks when placed in aqueous solutions may lead to the formation of large aggregates, especially at high polysaccharide concentration [24,73,123]. On top of that, the choice of method used may also serve as a major challenge, as each method is associated with its own advantages and limitations, and determines DDS properties such as shape, size distribution, porosity, and drug encapsulation efficiency [120]. In addition to identifying the ideal method based on the desired properties for the specific application, the methods that can be used also depend on properties of the drug, including solubility and surface properties, as well as the polysaccharide of choice, site of delivery, and biological activities.

The release of the kinetics of drugs from the delivery system can often be difficult to predict, especially for stimuli-responsive hydrogels, and are dependent on the degradation rate and swelling rate, both of which are influenced by both external factors and intrinsic delivery system properties [124,125]. Many researchers rely on the use of computational models such as zero-order kinetics, first-order kinetics, and Peppa’s model to predict the release of drugs from hydrogel DDSs. While these models may be accurate in some delivery systems, it is difficult to predict the release of drugs from unique and complex hydrogels as this is highly influenced by environmental factors such as pH, temperature, and the presence of chemicals, as well as the presence of hydrophobic or hydrophilic groups, the swelling capacity of the polysaccharide, and method of crosslinking used. In addition to computational models, due to the high cost of drugs, many researchers rely on model proteins or drugs such as BSA or dyes to predict release. While this allows the study to be more cost-effective, it may not always accurately represent or translate the in vitro findings to medicinal applications [126]. Additionally, even when accurately characterized during synthesis, hydrogel DDSs are prone to surface changes in response to external physiological stimuli, making the release pattern difficult to predict [120]. Other than release, the bioavailability of polysaccharide-based hydrogels may also be difficult to predict. In order to ensure the drug is released at the target site, the premature clearance of the DDS needs to be prevented [55]. Furthermore, the release time also needs to be taken into consideration when designing hydrogels to ensure sufficient time for drug release and accumulation prior to hydrogel clearance. From the various properties and limiting factors of DDS development it can be seen that the requirement to fine-tune methodology in order to achieve the desired properties is a major limitation associated with polysaccharide-based hydrogel DDSs.

## 6. Conclusions

The use of polysaccharides could enhance the targeted and controlled delivery of drugs to the target site due to their advantageous properties. These include high biocompatibility and biodegradability, easy availability, cost-effectiveness, and easy modification for overcoming limitations. Additionally, the presence of free functional groups such as carboxyl, amino, and hydroxyl groups allows for the chemical modification and drug conjugation. Polysaccharide-based hydrogels have been identified as having tunable physicochemical properties that make them ideal as drug carriers. Furthermore, due to their low immunogenicity, excellent biocompatibility, and biodegradation, they have been used for the development of in situ-forming, macroporous, and shear-thinning hydrogels. They can be used as hydrogel-forming microneedles for the delivery of accurate drug doses via transdermal administration and minimally invasive injections. The identification of such novel drug carriers potentially serves as a step towards more accurate dosing for sustainable treatments while also minimizing drug-associated cytotoxicity. There is a great amount of recent research carried out on the potential applications of various polysaccharide-based hydrogels, resulting in the identification of novel methodologies for the development of improved DDSs. However, the numerous requirements that need to be met for a DDS to be effective, as well as the factors that contribute towards its properties all need to be taken into consideration. This may serve as a potential challenge in the development of new DDS, as well as in the process of manufacturing DDSs on a large scale. Future research on micro/nanogel formation from natural polymers should have more research on the long-term cytotoxicity as well as an improvement of stability and control for in vivo applications.

## Figures and Tables

**Table 1:** Summary of various drugs and inhibitors delivered by polysaccharide-based hydrogels DDSs.

	Polymer Name	Polysaccharides and Additives	Delivered Molecule	Functions of Polysaccharides	Year	References
Genes	siRNA-loaded microgels	Cationic biodegradable dextran microgels	siRNA	Increase encapsulation amount, Controlled release of materials	2008	[74]
	Folate conjugated nanogel	Glycol chitosan-based nanogel	siRNA	Improve delivery of material	2013	[75]
	Plasmid DNA-loaded nanogel	CMC complex with bPEI nanogel	Plasmid DNA	Improve delivery of materials, Increase uptake and gene transfection	2015	[76]
	Double-network nanogels	Silane-crosslinked PEI with pH-responsive poly(2-(hexamethyleneimino)ethyl methacrylate)	DNA	pH-responsive delivery, Improve stability of genetic material	2019	[77]
	TAC6-derived nanogel	TAC6 polymer	PIAS1	Decrease gut inflammation by repressing NF-KB	2020	[78]
	Nanobody-functionalized nanogel	Nanobody conjugated DNA strands with Nb-DNA conjugate	miRNA	Increase accumulation of miRNA to desired site, Increase anti-tumor effect	2020	[79]
Peptides and Proteins	Hydrophillic thermo-responsive nanogels	NIPAM, poly(N-Isopropylmethacrylamide)	Protein	High protein encapsulation, Release protein at desired temperature	2019	[80]
	Nisin-loaded antimicrobial dual responsive nanogel	PLLA-g-CS	Peptide	Release nisin at specific pH and temperature	2019	[81]
	CytoC-embedded hydrogel	Acrylamide (AAm), 2-(dime- thylamino)ethyl methacrylate (DMAEMA) monomer and glycerol dimethacrylate (GDA)	Protein	Promoted delivery of CytoC to target sites, Improve tumor growth inhibition	2020	[82]
	pH and glucose dual-responsive nanogels	Dextran and poly(L-glutamic acid)-g-methoxy poly-(-ethylene glycol)/phenyl boronic acid (PLG-g-mPEG/PBA)	Protein	Mediates the delivery and release of protein	2019	[83]
	Temperature-sensitive nanogel	PEG, N-cyclopropylacrylamide (NCPAM), N-isopropylacrylamide (NIPAM), methacrylic acid (MAA) and trimethylsilylpropargylmethacrylate (TMSPMA)	Protein	Mediates the release of protein when triggered by enzyme and at specific temperature	2020	[84]
	Acid-degradable nanogels	Carboxymethyl chitosan	DOX	Decrease degradation of drug, improve cellular uptake of drug, improve stability of drug in physiological conditions	2019	[85]
Drugs	Hyaluronic acid nanogel	Hyaluronic acid, sucrose, glycerol diglycidyl ether (GDE)	[3-((E)-3-(4-hydroxyphenyl) acryloil)-2H-chromen-2-on	Improve delivery of drug, improve long-term delivery of drug	2019	[86]
	Zwitterionic phosphorylcholine-based nanogel	Poly(phosphorylcholine) (HPMPC)	DOX	Longer blood circulation of nanogel, improve tumor inhibition, effective degradability to release drug to desired site	2021	[87]
	Zwitterionic phosphorylcholine-based nanogel Ca2+ responsive deacetylated gellan gum based in situ-nanogel	Poly(phosphorylcholine) (HPMPC) Deacetylated gellan gum (DGG)	DOX Harmine nanocrystals (HAR-NC)	Able to deliver drugs passing through BBB, release drug in hypoxic environment	2021	[88]
				Improve intranasal drug delivery, increase bioavailability of drug in brain,	2021	[89]
	Chitosan-polypyrrole nanogels	Chitosan, pyrrole	DOX	Highly adaptive to surrounding pH, compatible at physiological pH, high loading of drug, prolonged circulation, increase tumor accumulation of nanogel	2021	[90]
	Tumor microenvironment (TME)-responsive P(CPT-MAA) prodrug nanogel	Methacrylic acid (MAA), CPT monomer (CTPM), and N-N’-methylenebisacrylamide (Bis)	Campthothecin (CPT)	Improve drug delivery, releases drug at specific conditions	2019	[91]
	Supramolecular polymer nanogel	Hyperbranced polyglycerol, polyglycerylamine, carboxylic-substituted copillar[5]arene, carbonyldiimidazole, sodium 6-chlorohexyl sulfate, DMF and triethylamine	Dexamethasone	Increase drug loading capacity, improve skin penetration	2019	[92]
	Hybrid bovine serum albumin-gum arabic aldehyde (BSA-GAA) nanogels	Gum arabic aldehyde, bovine serum albumin	5-FU	Increase drug release at acidic pH, no toxicity of drug when loaded into nanogel	2021	[93]

## Data Availability

Not applicable.
